# Therapeutic effect and metabolomics mechanism of *Patrinia Villosa* (Thunb.) juss on liver injury in rats

**DOI:** 10.3389/fphar.2022.1058587

**Published:** 2022-10-21

**Authors:** Li-Man Qiao, Hui Zhang, Wei Liu, Dan Lou

**Affiliations:** ^1^ The Second Affiliated Hospital and Yuying Children’s Hospital of Wenzhou Medical University, Wenzhou, China; ^2^ School of Pharmaceutical Science, Liaoning University, Shenyang, China

**Keywords:** *Patrinia villosa* (Thunb.) juss, liver injury, inflammation, metabolism, UPLC-MS

## Abstract

Patrinia villosa (Thunb.) Juss (P.V) is widely used in the treatment of chronic diseases, such as appendicitis, enteritis and gynecological inflammation. Modern research indicated that the herb has pharmacological effect on liver injury caused by inflammation, but the metabolomics mechanism is not clear. For the purpose of discovering the therapeutic effect and metabolomic mechanism of P.V on liver injury, 40 Sprague-Dawley (SD) rats were divided into normal group, model group, and P.V groups (0.98, 1.97, and 2.96 g/kg). The model group and P.V groups were injected intraperitoneally with 40% CCl_4_ (v/v, olive oil) to establish liver injury model. After administration of P.V for seven consecutive days. Therapeutic effect of P.V on liver injury rats were analyzed. P.V could decrease serum alanine aminotransferase (ALT) and aspartate aminotransferase (AST) levels of liver injury rats as a dose-dependent manner. Compared with the model group, the pathological analysis of liver tissue of P.V groups exhibit significant decrease tendency of hepatic tissue structure destruction, cytoplasmic vacuolation, cellular swelling, and inflammatory cell infiltration as a dose-dependent manner. 82 endogenous metabolites in rat serum and liver were analyzed by Ultra-high performance liquid chromatography tandem mass spectrometry (UPLC-MS/MS). 14 metabolites in serum and 26 metabolites in liver were significantly different between the P.V group (2.96 g/kg) and the model group. Metabolic pathway analysis revealed that the main pathway including alanine, aspartate and glutamate metabolism, and TCA cycle were significantly altered. It is suggested that P.V can alleviate CCl_4_ induced liver injury, and its effect on metabolites may be an important mechanism of action.

## 1 Introduction

The liver is an important metabolic organ of the human body. It mainly has the physiological functions of storing glycogen, decomposing blood sugar, and participating in the metabolism of protein, fat, vitamins and hormones (Wu et al., 2017a). The liver not only regulates metabolism, but also has the function of detoxification, phagocytosis and defense against foreign toxic substances (Mega et al., 2021; Qian et al., 2021). The extensive application of toxic chemicals, drugs, alcohol, dietary supplements and food additives may cause liver injury (Yang et al., 2017). Liver cell injury will cause inflammatory reaction, oxidative stress, fat-like changes, and then a series of pathological changes such as liver cell necrosis, apoptosis and fibrosis (Navarro et al., 2017; Kong et al., 2021).

Liver injury is progresses to the stage of liver failure, which has clinical manifestations such as fatigue, anorexia, abdominal fullness and loose stools (Wang et al., 2021). It is often delayed due to insufficient attention, and severe cases can even cause liver failure and endanger life (Jaeschke et al., 2012). Poor prognosis after liver injury have always been a problem in the medical community (Khemichian et al., 2021). In modern medicine, symptomatic treatment is often used clinically to treat liver injury (Navarro et al., 2017). At the same time of treatment, it lacks comprehensive consideration of systemic pathological state, which will cause harm to the body (Sun et al., 2014).

*Patrinia villosa* (Thunb.) Juss (P.V) is a traditional Chinese medicine which is commonly used in the treatment of chronic inflammation diseases, such as intestinal carbuncle, pulmonary carbuncle, dysentery, postpartum petechial abdominal pain, and carbuncle (Huang et al., 2022). Modern medical research has found that the herb has various pharmacological activities such as anti-inflammatory, sedative and hypnotic, anti-viral, anti-tumor, liver-protecting, and immunity-enhancing actions (Zheng et al., 2012; Li et al., 2016; Sheng et al., 2019), but its mechanism of liver-protection action has not been reported.

Chemical investigation indicated that saponins, flavonoids, coumarins, cycloethenes terpenoids, lignans, and volatile oils are the main active ingredients of P.V (Peng et al., 2005; Xiang et al., 2016; Xin-Jia et al., 2016; Yang et al., 2016). P.V can inhibit the hepatitis virus, improve liver function, and promote hepatocyte regeneration and bile secretion (Liao et al., 2012; Dai et al., 2013). Previous study indicated apigenin in P.V could inhibit hepatocellular carcinoma cell growth by CyclinD1/CDK4 regulation *via* p38 MAPK-p21 signaling (Li et al., 2016). In this study, the therapeutic effect of P.V on liver injury was studied, and the metabolic mechanism was analyzed by UPLC-MS/MS.

## 2 Materials and methods

### 2.1 Materials and reagents

*Patrinia villosa* (Thunb.) Juss was obtained from Xinqi Traditional Chinese Medicine Pellets Co., Ltd (Hebei, China), and the herb was identified by Dr. Jian Wu from Harbin University of Commerce. A voucher specimen (No. PVJ20130821) was deposited at the Pharmacognosy Laboratory of Liaoning University. L-tryptophan (Try), L-kynurenine (Kyn), Oxidizided glutathione (GSSG), N-phenylacetylglycine (N-Phe), 5-hydroxytryptophan (5-HTP), L-leucine (Leu), 5-HT, cholic acid (CA), and Glutathione (GSH) were obtained from Dalian Melon Biology Technology Co., N-Ethylmaleimide (NEM) and formic acid were purchased from Sigma-Aldrich Chemical Co., PBS buffer was obtained from Hyclone Chemical Co., Internal standard (L-phenylalanine-*d*_5_, *d*_5_-Phe) was obtained from Cambridge isotope laboratories. All standard stock solutions were stored at -40 °C before use. Methanol and acetonitrile were purchased from ACS Chemical Co.

### 2.2 Preparation of *Patrinia villosa* (Thunb.) Juss extract

960 g of P.V was refluxed with 9.6 L of distilled water for 1.5 h, before being cooled, the supernatant was filtered and concentrated under reduced pressure gave extract residue of 90.29 g. Samples were stored at 4°C in a refrigerator before use.

### 2.3 Animals and treatments

A total of 40 male Sprague-Dawley rats, weighing about 200 ± 20 g, were divided into five groups (*n* = 8) including normal group, model group and P.V groups with three dosages (0.98, 1.97, and 2.96 g/kg). After 2 weeks of adaptive feeding, the P.V groups and model group were injected intraperitoneally with 40% CCl_4_ (v/v, olive oil) at the dose 2 ml/kg (Wang et al., 2021). P.V groups were intragastric administered once daily for 7 days, the normal group and model group were intragastric administered with the same volume of saline once daily for 7 days. All the rats were anesthetized with sodium pentobarbital intraperitoneally 2 h after administration on the seventh day and blood samples were collected from the abdominal aorta and exposed to laparotomy. Blood was collected in a non-anticoagulant tube, and serum was collected after centrifugation at 3,500 rpm and 4 °C for 10 min. Rat livers were washed with normal saline after routine sampling and then fixed in formalin solution for the preparation of pathological sections of liver tissue. The histopathological changes in the rat livers were observed by microscope after HE staining.

### 2.4 Metabolic analysis

Metabolic analysis was performed according to the previous report with minor modification (Wang et al., 2022). In this study, 82 endogenous metabolites involved in oxidative stress, inflammation, amino acid metabolism, purine metabolism, the tricarboxylic acid cycle, glycolysis, and lipid metabolism were selected as targets.

For the preparation of stripped serum, 1.8 g of activated carbon powder was added to 30 ml of rat serum. After mixing in a shaker for 2 h, the mixture was centrifuged at 13,500 rpm for 20 min at 4°C. The supernatant was collected and filtered with Millipore express PES membranes (Merck Millipore, Ltd.) in order of 5, 1.2, 0.45 and 0.22 μm, respectively, to prepare the stripped serum samples.

For the preparation of standard solutions, L-tryptophan (Try), L-kynurenine (Kyn), oxidizided glutathione (GSSG), N-phenylacetylglycine (N-Phe), 5-hydroxytryptophan (5-HTP), L-leucine (Leu), 5-HT and cholic acid (CA) were dissolved in methanol and prepared to a final concentration of 1 mg/ml. Glutathione (GSH) was dissolved in 10 mM of NEM PBS buffer to a final concentration of 0.5 mg/ml. All standard stock solutions were stored at -40°C and protected from light. L-phenylalanine-*d*_5_ (*d*_5_-Phe) was used as the internal standard, and a 10 ng/ml stock solution was prepared in methanol and stored at –40°C. The components were mixed, and distilled water was used to prepare standard curve solutions and diluted with 50% methanol-water to prepare a series of standard solutions. Because of the degradability of the GSH component, to ensure its quantitative assay accuracy and its component stability, GSH was derivatized with the reaction of NEM and GSH at an NEM concentration of 50 mM (Carretero et al., 2014; Younossi et al., 2016; Wang et al., 2022).

200 μL of PBS solution containing 10 mM of NEM and 1,200 μL of methanol (containing 10 ng/ml of *d*_5_-Phe) was added to 200 μL of serum in the tube and incubated at -20°C for 20 min. After vortex mixing, centrifugation was performed at 13,500 rpm and 4°C for 15 min 1,000 μL of supernatant were collected and blown dry by nitrogen to obtain samples. Samples were reconstituted with 50 μL of 5% acetonitrile (ACN) in water and centrifuged at 13,500 rpm and 4°C for 15 min. Then, 30 μL of the supernatant was pipetted into a sample tube for metabolic analysis.

0.2 g of liver tissue was weighted precisely, and 10 times the volume of normal saline were added in a centrifuge tube and homogenized, then the samples were centrifuged at 12,000 rpm for 15 min 100 μL of PBS solution containing 10 mM of NEM and 800 μL of methanol (containing 10 ng/ml of *d*_*5*_-Phe) was added to 100 μL of supernatant in the tube and incubated at -20°C for 20 min. After vortex mixing, centrifugation was performed at 13,500 rpm and 4°C for 15 min 800 μL of supernatant were collected and blown dry by nitrogen to obtain samples. Samples were reconstituted with 50 μL of 5% acetonitrile (ACN) in water and centrifuged at 13,500 rpm and 4°C for 15 min. Then, 30 μL of the supernatant was pipetted into a sample tube for metabolic analysis.

Metabolic analysis was performed on an AB4000Q TRAP LC/MS/MS mass spectrometer with deuterated internal standards (IS) (Phe-*d*_5_) as the internal standard. The column used was Waters X BridgeTM BEH C18 2.5 μm, 3.0 × 100 mm. Positive and negative ions were quantified and semi-quantified using an ESI ion source detector. UPLC was performed using 0.1% formic acid-water (A) and acetonitrile (B) as elution systems. The gradient separation conditions were 0–2 min, 5% (B); 2–5 min, 5–50% (B); 5–6 min, 50% (B); 6–17 min, 50–95% (B); 17–18 min, and 95%–5% (B). The injection volume was 10 μL and flow rate was 0.3 ml/min. Sample detecting were in an MRM mode with positive and negative model. Curtain gas (CUR): 10; collision gas (CAD): Medium; ion spray voltage: The Ion Spray voltage: ±4500V; Temperature: 450°C; ion source gas (GS1): 40; ion source gas (GS2): 40.

#### 2.4.1 Methodology validation

Methodology validation was conducted in accordance with the “Guidance for Industry-Bio analysis Method Validation” (Food and Drug Administration, September 2013) issued by the United States Food and Drug Administration. A mixed standard solution of GSH, L-tryptophan, L-kynurenine, GSSG, N-phenylacetylglycine, 5-HTP, L-leucine, 5-HT, and cholic acid was prepared and diluted successively to prepare two solutions at each concentration. The standard curve was calculated by the ratio of the peak area of the sample to the area of the internal standard sample, and the least-squares method with 1/x weight were used to fit the linear regression equation of the standard curve.

#### 2.4.2 Precision and accuracy assay

The assay precision of the UPLC-MS method was analyzed using quality control (QC) samples, and the QC samples were assayed using untreated rat serum to determine the intraday and interday precision.

High, medium and low concentration QC samples were prepared by adding standard solutions of L-tryptophan, L-kynurenine, GSSG, N-phenylacetylglycine, 5-HTP, L-leucine, 5-HT, cholic acid, and GSH-NEM to stripped plasma. The stability of the method was evaluated by calculating the sample concentration according to the sample test content calculation method.

#### 2.4.3 Data analysis

The MS analysis was performed by the software of Analyst (1.6.4). The standard curve method was used to measure the ratio of the peak area (Area ratio) of the sample to the internal standard. The least-squares method with 1/x weight was used to fit the standard curve linear regression equation. For test metabolites where no standard exists, a semi-quantitative analysis was performed to evaluate the change trend of the same test component based on the ratio of the peak area of the test sample to the area of the internal standard.

#### 2.4.4 Metabolic pathway analysis

Pattern discrimination analysis was performed on each group of relevant data using the principal component analysis (PCA) method with SIMCA-P software. Based on the differential metabolites, serum metabolite profiles were analyzed using the The Human Metabolome Database (HMDB) and Kyoto Encyclopedia of Genes and Genomes (KEGG)-related metabolomics databases to identify the metabolic pathways associated with the prevention of CCl_4_-induced liver injury in rats.

### 2.5 Statistics analysis

The statistical analysis was performed using SPSS 17.0. ALT, AST, and the metabolites contents were described by means and standard deviations (Mean ± SD). ALT and AST levels were compared between groups by t-tests. *p* < 0.05 was taken as a statistically significant.

## 3 Results

### 3.1 Protect effect of P.V on liver injury

#### 3.1.1 Histopathological analysis

In terms of the gross anatomical appearance, the adhesion condition of the liver to peritoneum and intestine was severe in the model group, no adhesion condition was found in the normal group, and the adhesion degree was lower in the P.V groups.

**FIGURE 1:**
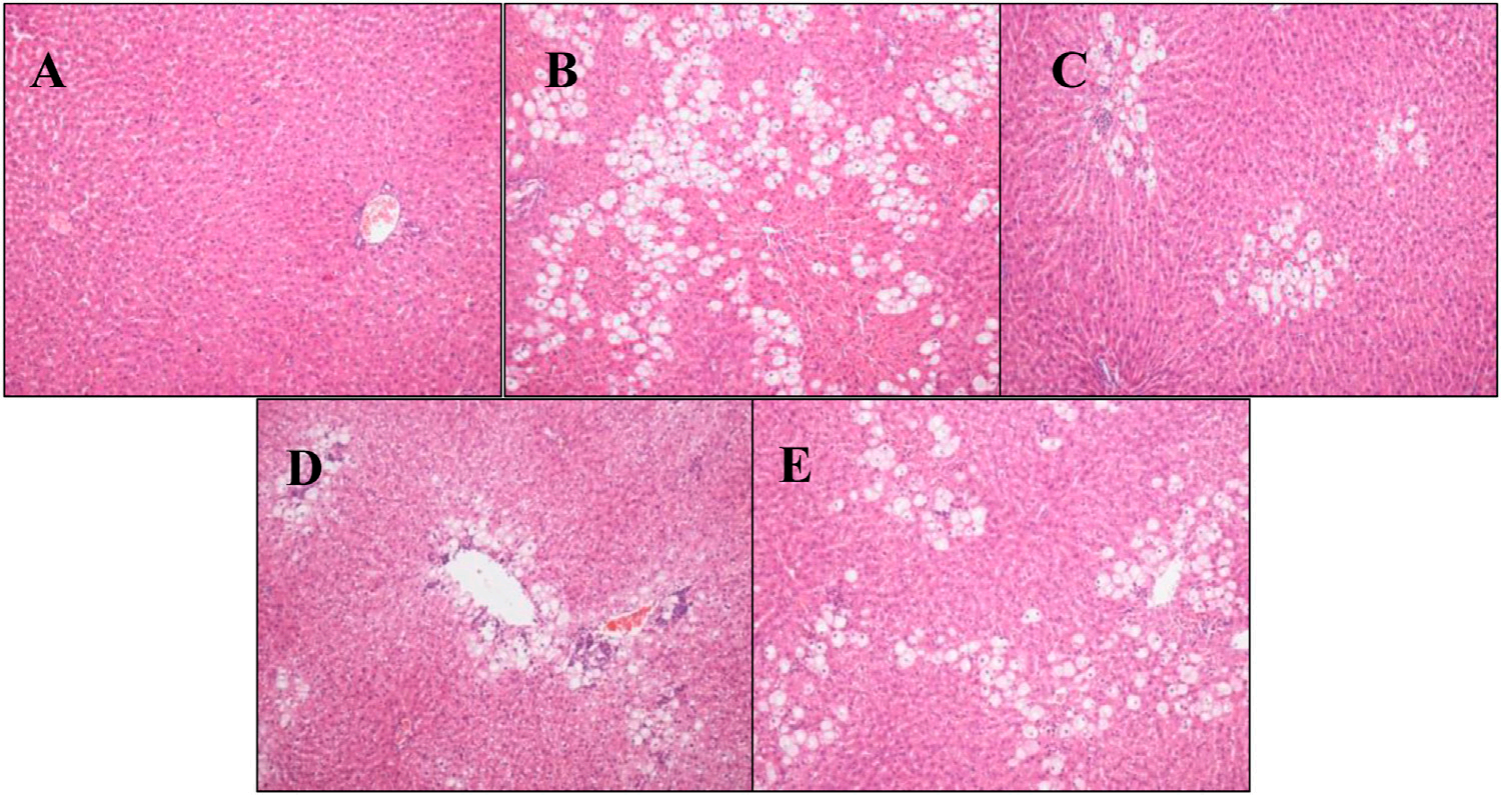
Pathological analysis results of rat liver tissue (H-E staining, × 100) **(A)** Blank group **(B)** Model group **(C)** P.V group (2.96 g/kg) **(D)** P.V group (1.97 g/kg) **(E)** P.V group (0.98 g/kg).

Pathological analysis of rat liver tissue sections showed that the structure of rat liver tissues in the normal group was intact and clear, the cell morphology was regular, and no necrosis and inflammatory cell infiltration were observed. In the model group, the liver tissue structure was severely damaged, the cells were swollen and denatured, cytoplasmic vacuolation was observed, and inflammatory cell infiltration was observed to different degrees. Compared with the model group, hepatic tissue structure destruction, cytoplasmic vacuolation, and cellular swelling significantly decreased, and inflammatory cell infiltration decreased, in the P.V groups. The injury level tended to decrease as the dose dependant manner (Figure 1), which was consistent with the changes in ALT and AST levels.

#### 3.1.2 ALT and AST in serum

**FIGURE 2:**
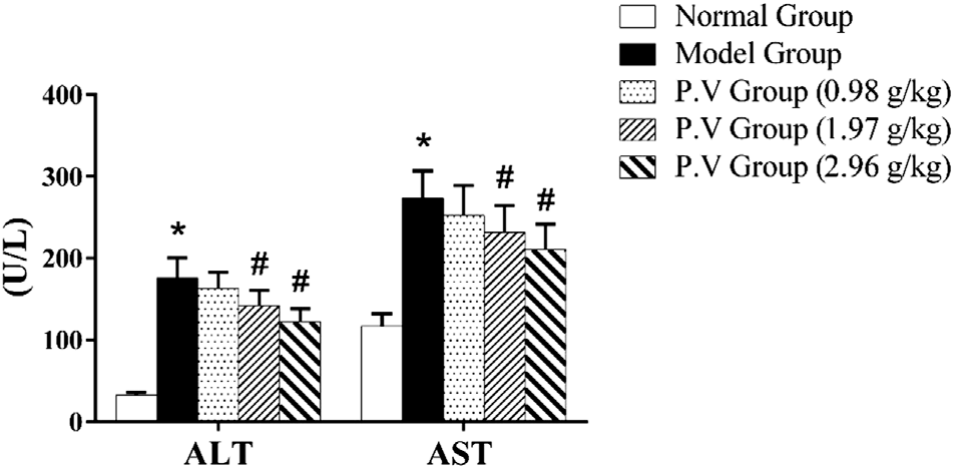
ALT and AST levels in serum. *p <0.05, model group vs normal group; #*p* <0.05, P.V group vs. Model group.

Compared with the normal group, the levels of ALT and AST in the model group were significantly higher (*p* < 0.01), indicating that the model of CCl_4_-induced liver injury was successful. The levels of ALT and AST in P.V groups decreased compared with those in the model group as a dose-dependent manner, indicating that P.V could reduce the levels of ALT and AST in the serum of rats with liver injury to a different extent and play a liver-protective role in liver injury (Figure 2).

### 3.2 Metabolic analysis

#### 3.2.1 Linearity

The linear regression equation of the standard curve was fitted using the least-squares method and 1/x weighting, using the concentration of the metabolites as the horizontal coordinate and the ratio of the peak area of the detected component to the internal standard component as the vertical coordinate (Table 1).

**TABLE 1:** Calibration curves of the nine metabolites.

Metabolites	Calibration curve	R^2^	Linearity range
GSH	Y = 0.0001X—0.0065	0.9995	100–10000 ng/ml
GSSG	Y = 0.0022X + 0.3209	0.9981	200–20000 ng/ml
L-Leucine	Y = 0.0279X + 2.4351	0.9983	100–10000 ng/ml
L-Kynurenine	Y = 0.0347 X + 0.0147	0.9980	5–500 ng/ml
L-Tryptophan	Y = 0.0017 X + 0.8435	0.9921	600–60000 ng/ml
5-HTP	Y = 0.0891X - 0.0088	0.9919	0.2–20 ng/ml
Cholic acid	Y = 0.00005 X - 0.0009	0.9932	40–4000 ng/ml
5-HT	Y = 0.0505 X + 0.001	0.9986	0.8–80 ng/ml
N-phenylacetylglycine	Y = 0.0154X + 4.1334	0.9932	250–25000 ng/ml

#### 3.2.2 Accuracy and precision

The results indicate that the accuracy and precision of the method was appropriate for all study samples. Precision analysis of GSH, GSSG, L-Leu, L-Try, L-Kyn, 5-HTP, cholic acid, 5-HT, and N-phenylacetylglycine indicated that the intra-day precision of each metabolite ranged from 2.35% to 4.19%, and the inter-day precision ranged from 2.76% to 4.82%. All the values of these parameters were less than 5% which had satisfactory results within the acceptable criteria.

#### 3.2.3 UPLC-MS/MS analysis

**FIGURE 3:**
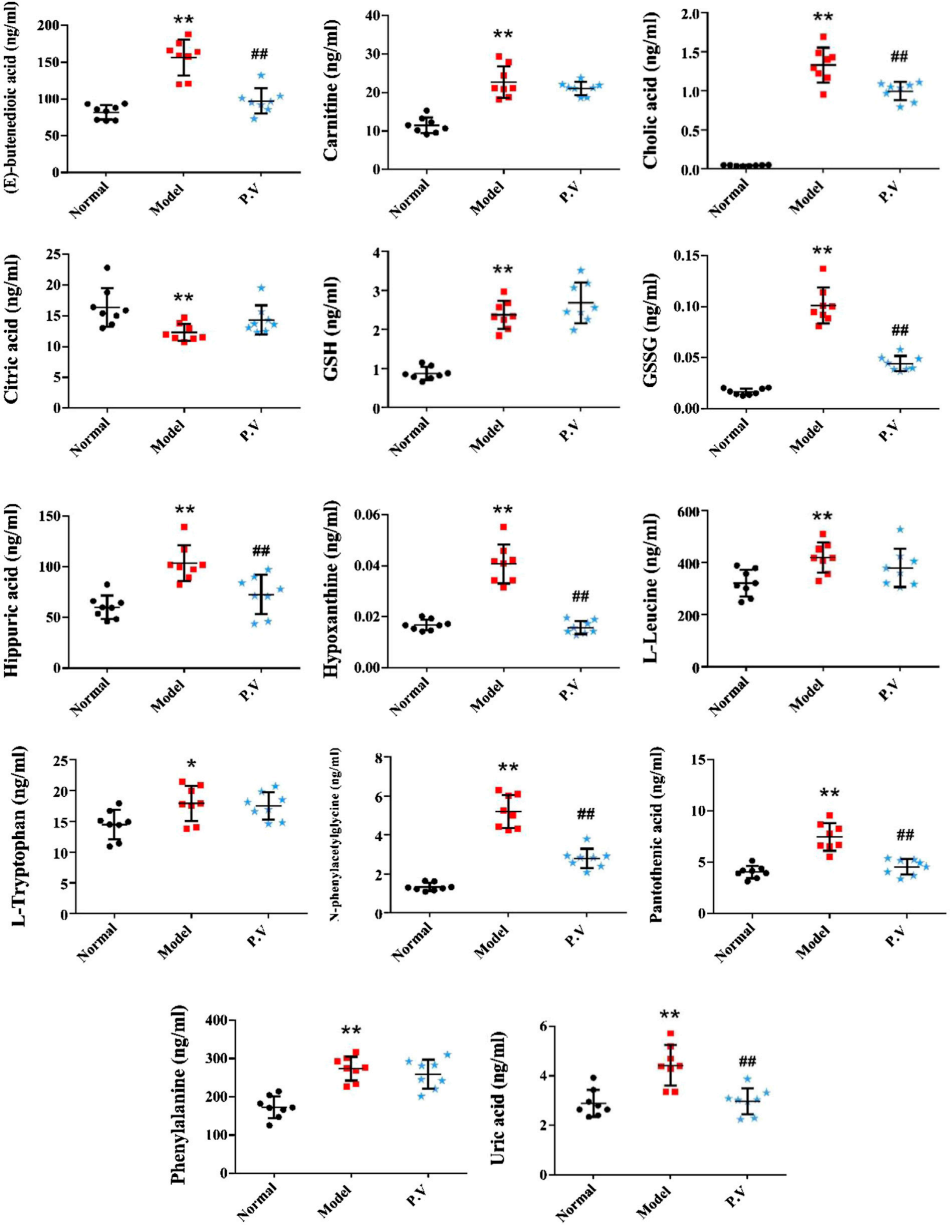
Effect of P.V on metabolites in serum of rats with liver injury (*n* = 8, mean ± SD).

**FIGURE 4:**
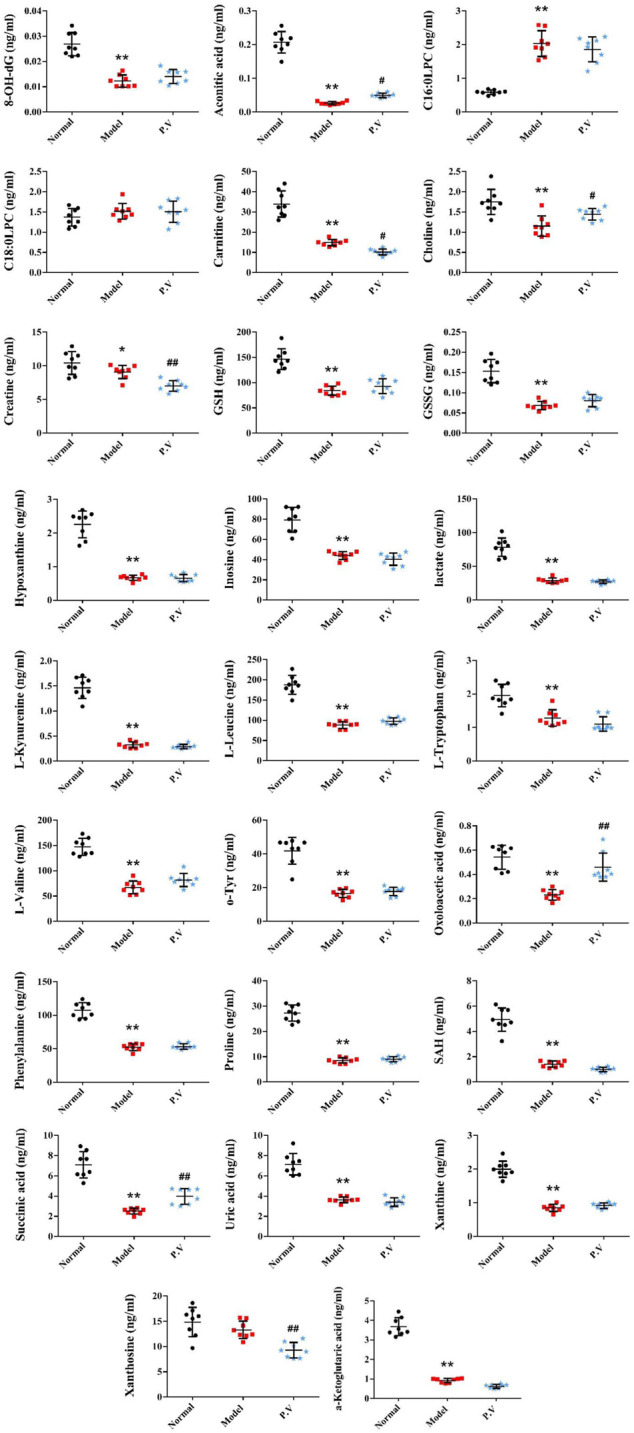
Effect of P.V on metabolites in liver of rats with liver injury (*n* = 8, mean ± SD).

On the basis of establishing a stable and reliable method for the determination of metabolites by UPLC-MS/MS, 82 metabolites were simultaneously determined in normal group, model group, and P.V group (2.96 g/kg). We then compared the differences of 14 metabolites in serum (Figure 3) and 26 metabolites in liver (Figure 4) between the groups by combining the chromatograms with the relative standard deviation (RSD%) of the content of each component within the same group, which was less than 30%. The results indicated that there were significant changes in metabolites in the serum samples of the model group, and there were multiple related differential metabolites that could be differentiated between the normal group and the model group. The GSH/GSSG ratio decreased significantly in the model group compared with that in normal group, which was consistent with the decrease in the antioxidant capacity in the model group. The levels of phenylalanine and citric acid in the model group were significantly lower than those in the normal group, and the levels of phenylalanine and citric acid in the model group recovered to the level of the normal group after treatment. The levels of L-leucine, cholic acid, N-phenylacetylglycine, hippuric acid, carnitine, and hypoxanthine in the model group were significantly higher than those in the normal group.

#### 3.2.4 Pathway analysis

**FIGURE 5:**
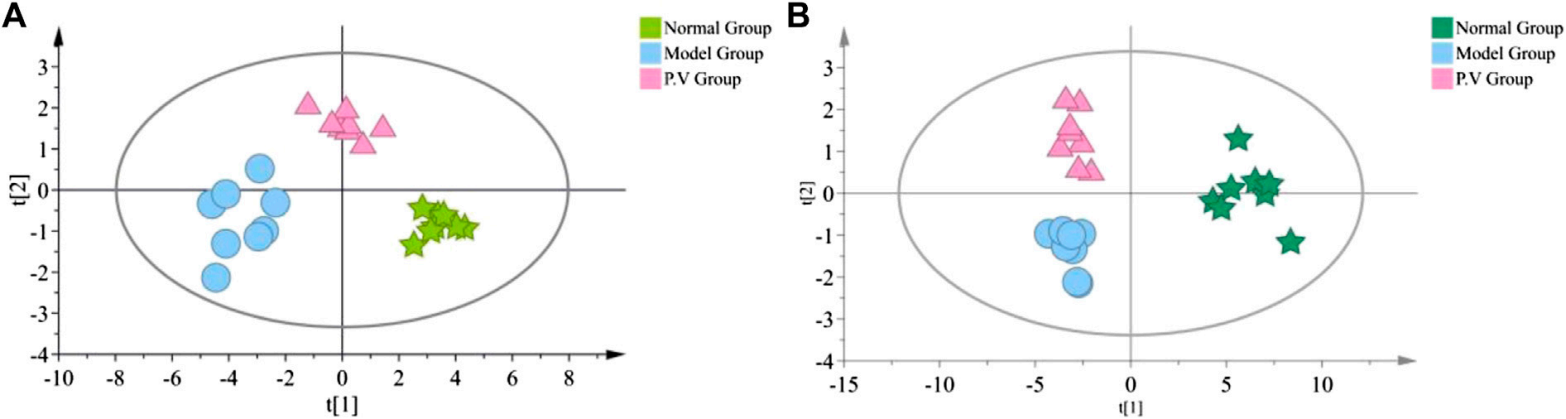
PCA analysis of metabolites **(A)** Serum **(B)** liver.

**FIGURE 6:**
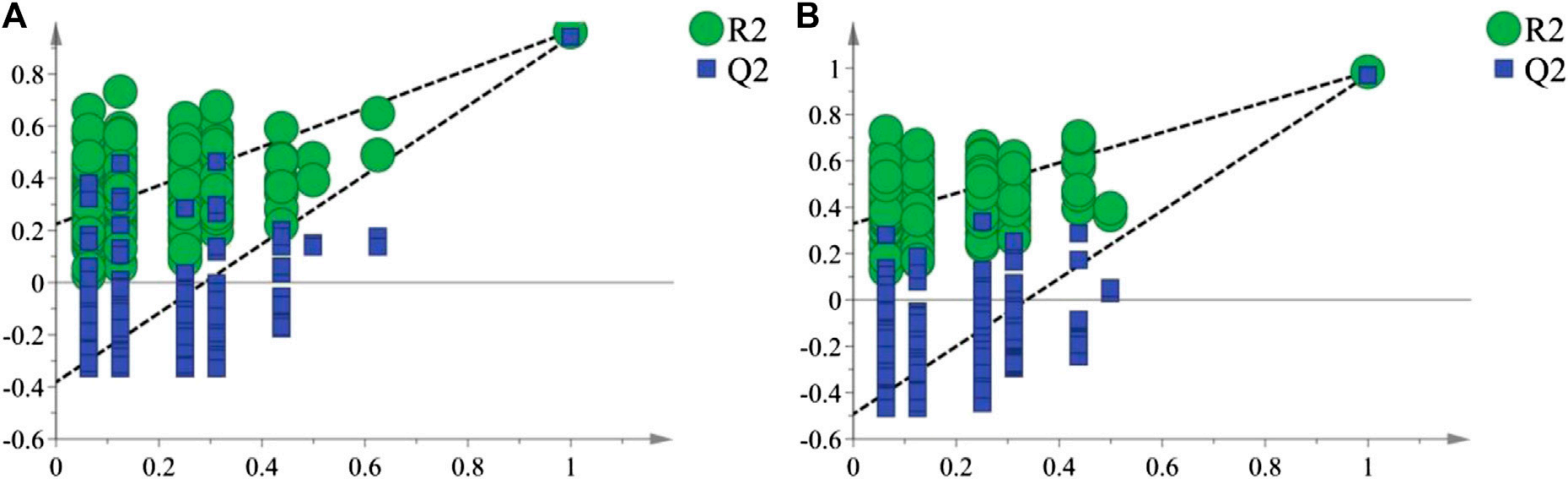
P-test analysis of the metabolites **(A)** Serum **(B)** liver.

As shown in the PCA analysis (Figure 5), the serum samples (Figure 5A) and liver samples (Figure 5B) of the normal group and model group were distributed in different regions and far away from each other, and the same group showed an obvious aggregation trend, indicating the difference in metabolite content between the normal group and model group. This further confirmed the success of the model of liver injury and also suggested that P.V could significantly adjust the biomarker content in the serum and liver of rats with liver injury, causing an obvious change in the metabolic network. P-test analysis also showed that the different components tested were replaced to ensure that there was no overfitting in the between-group separation models (Figure 6).

**FIGURE 7:**
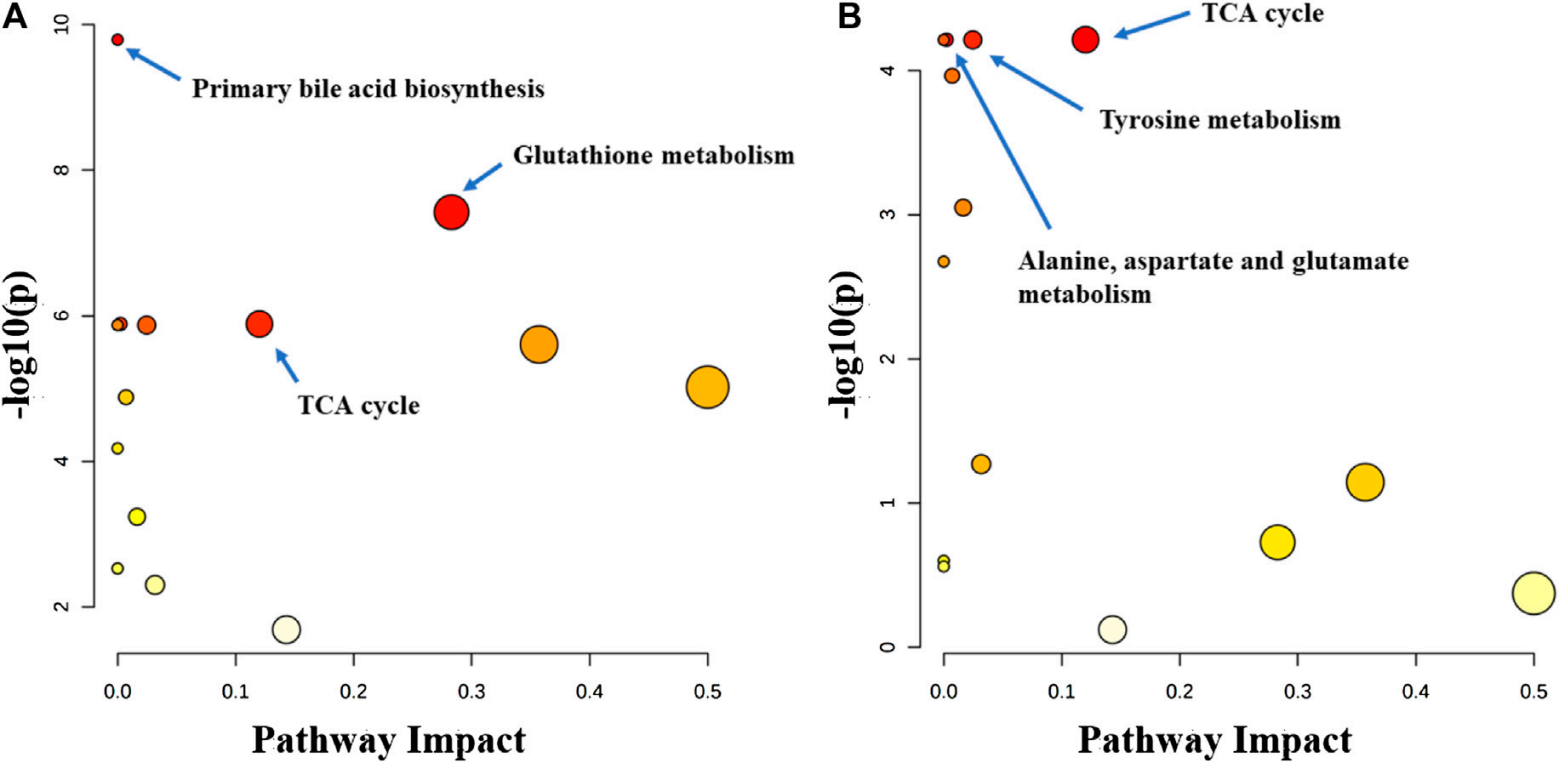
Metabolic pathway analysis of serum in rats **(A)** Normal group vs. Model group **(B)** P.V group vs. Model group.

**FIGURE 8:**
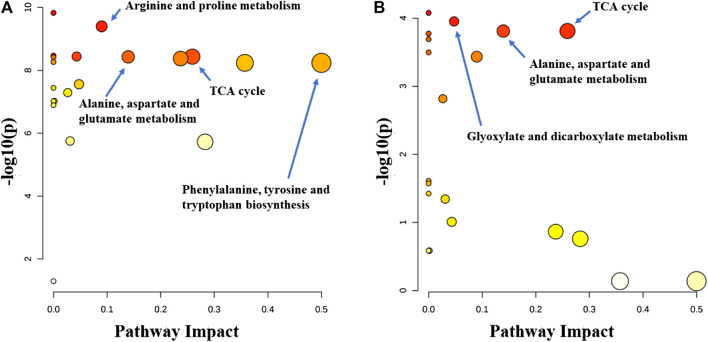
Metabolic pathway analysis of liver in rats **(A)** Normal group vs. Model group **(B)** P.V group vs. Model group.

Metabolitic pathway analysis indicated that primary bile acid biosynthesis, glutathione metabolism, and TCA cycle were the most important metabolic pathway affected in the serum of the normal group vs. model group (Figure 7A). The alanine, aspartate and glutamate metabolism, TCA cycle, and tyrosine metabolism pathways were significantly affected by the administration of the P.V (Figure 7B). While in the liver tissue, arginine and proline metabolism, TCA cycle, alaine, aspartate and glutamate metabolism, Phenylalanine, tyrosine and tryptophan biosynthesis were the most important metabolic pathway affected in the normal group vs. model group (Figure 8A). Alanine, aspartate and glutamate metabolism, TCA cycle, glyoxylate and dicarboxylate metabolism pathways were significantly affected by the administration of P.V (Figure 8B).

## 4 Discussion

In this study, the liver injury induced by CCl_4_ was improved by P.V. Further, the elevation of serum ALT and AST in the rat model was reversed, the liver histological damage was alleviated to a certain extent, and the effect showed a dose-dependent manner. 82 molecules related to oxidative stress, inflammation, amino acid metabolism, purine metabolism, tricarboxylic acid cycle, glycolysis, and lipid metabolism were analyzed. It was found that 14 metabolites in serum and 26 metabolites in liver showed significant changes, of which appeared to be key different metabolizers.

It is well known that despite having different etiologies, liver injuries are frequently associated with excessive oxidative stress. Liver function is protected by enhancing the activity of the endogenous antioxidant defense system (Wang et al., 2017). Glutathione has two active forms, reduced and oxidized. GSH is an important scavenger to protect against oxidative stress in the liver. A decreased GSH/GSSG ratio is used as the index of oxidative stress. In this study, the serum GSH/GSSG values in the model group (23.7) were significantly decreased compared with those in the normal group (51.2), which is in accordance with the state of abrupt decrease of antioxidant capacity in the rat model of liver injury. The GSH/GSSG value in the P.V group (60.1) was similar to that in the normal group, suggesting that P.V could repair the liver injury by reducing the level of oxidative stress in the hepatocytes.

Nucleoside is the major component of nucleic acid, while hypoxanthine is a metabolite of nucleoside and is an important alkaloid purine. AMP first generates hypoxanthine, which is oxidized to xanthine under the action of xanthine oxidase and ultimately decomposes to form uric acid, which is excreted in the urine. This pathway is an important metabolic pathway in the body, and gout is a clinical condition caused by abnormal uric acid metabolism. In this study, the increase of hypoxanthine content in the serum of model rats is considered to be related to a decrease in xanthine oxidase activity and the hindrance of hypoxanthine oxidation.

The TCA cycle is a common pathway for the metabolism of the three major nutrients: sugar, fat and amino acids. Citric acid is the key intermediate product of the TCA cycle, and oxaloacetic acid and acetyl-CoA are the important regulatory points of the TCA cycle. In this study, citric acid levels in serum were significantly reduced in the model group, presumably due to impairment of the TCA cycle process in rat hepatocytes (Li et al., 2014; Wu et al., 2017b), partial recovery of the TCA cycle, and increased citric acid levels after treatment.

Amino acid metabolism can be used to synthesize specific proteins, peptides, and nitrogen-containing compounds, either through decarboxylation by deamination, transamination, and catabolism of ammonia, or by the release of energy through citric acid cycling. Therefore, these changes in amino acid metabolism may elicit important signaling events within hepatocytes. Our results suggest that P.V can cause metabolic abnormalities of amino acid. L-Leucine is one of the eight essential amino acids in the human body. It has been reported that CCl_4_ can cause decreases in leucine, glutamine/glutathione, and betaine contents, presumably due to inhibition of the expression of these amino acid synthesis genes (Li et al., 2014). Phenylacetylglycine could be involved in the metabolism of intestinal microorganisms. It has been suggested that the level of phenylacetylglycine in the urine of CCl_4_ liver injury model rats decreased (Wu et al., 2017b). In this study, the level of N-phenylacetylglycine in the serum of the model group increased and the level of acetylglycine in the P.V group decreased. We presume that this was related to the decrease in urinary excretion and the increase in the serum concentration in the model rats.

In this study, the therapeutic effect of P.V on liver injury was assayed. P.V could decrease the hepatic tissue structure destruction, cytoplasmic vacuolation, cellular swelling, and inflammatory cell infiltration obviously. And it could decrease ALT and AST levels in rats suffered liver injury as a dose dependant manner. Metabolic mechanism analysis revealed that P.V could treat liver injury by regulating alanine, aspartate and glutamate metabolism, and TCA cycle.

## Data Availability

The raw data supporting the conclusions of this article will be made available by the authors, without undue reservation.
